# Switching to Atripla (EFV/FTC/TDF) from Kivexa (ABC/3TC) plus EFV leads to improved perceptions of treatment: results from the ROCKET 1 study

**DOI:** 10.1186/1758-2652-13-S4-P42

**Published:** 2010-11-08

**Authors:** V Cooper, R Horne, J Ewan

**Affiliations:** 1 The School of Pharmacy, University of London, London, UK; 2 Gilead Sciences, Cambridge, UK

## Purpose of the study

The aim of this analysis was to examine the impact of switching to ATR from KVX+EFV on patients perceptions of treatment including the following: treatment intrusiveness, treatment satisfaction and HIV/HAART related symptom experiences; self-reported adherence was also assessed.

## Methods

Data was collected as part of ROCKET 1, a Phase 4, open-label, randomised, UK, multicentre, controlled study. At baseline 159 subjects stable on KVX+EFV with raised cholesterol were randomized 1:1 to one of two treatment groups: Treatment Group 1 (TG1): switch to ATR; Treatment Group 2 (TG2): continuation of previous stable regimen of KVX+EFV. At week 12, subjects in TG2 were switched to ATR. Treatment in both groups continued to study week 24. Subjects completed questionnaires assessing treatment intrusiveness, satisfaction with treatment, and symptom experiences at baseline and at weeks 4, 12, 16, and 24. Adherence over the preceding 30 days was measured using a visual analogue scale.

## Summary of results

Following the switch to ATR, 69% of subjects in TG1 indicated that they preferred their current regimen to their previous antiretroviral regimen. At week 12, between group comparisons showed those who switched to ATR perceived their treatment to be more convenient (p<0.05) and tolerable (p<0.05), reported fewer symptoms (p<0.0001) and were less bothered by side-effects (p<0.005) than those who remained on KVX+EFV. There was no difference between groups in reported adherence: 87% of those who switched to ATR and 86% of those who remained on KVX+EFV reported taking at least 95% of their antiretroviral medicines over the past 30 days (p>0.05). Those who switched to ATR were more likely than those who remained on KVX+EFV to experience a reduction in symptoms (p<0.0001) and treatment intrusiveness (p<0.05) between baseline and week 12.

## Conclusions

These findings indicate that switching to ATR from KVX+EFV leads to improved perceptions of treatment among people who have raised cholesterol. These findings complement those from the primary analysis of ROCKET 1 showing improvements in lipid profiles among subjects who switched from KVX+EFV to ATR.

